# The overexpression of Tipe2 in CRC cells suppresses survival while endogenous Tipe2 accelerates AOM/DSS induced-tumor initiation

**DOI:** 10.1038/s41419-021-04289-0

**Published:** 2021-10-26

**Authors:** Yan Li, Na Zhang, Chao Ma, Wenwen Xu, Guiyuan Jin, Yi Zheng, Lei Zhang, Bingyu Liu, Chengjiang Gao, Suxia Liu

**Affiliations:** 1 Key Laboratory of Infection and Immunity of Shandong Province, Department of Immunology, Shandong University School of Basic Medical Science, Jinan, Shandong China; 2 Department of Blood Transfusion, Qilu Hospital of Shandong University, Ji’nan, People’s Republic of China; 3 Department of Pathology, Shandong University School of Basic Medical Science, Ji’nan, People’s Republic of China

**Keywords:** Colon cancer, Immunosurveillance

## Abstract

Aging is a natural and progressive process characterized by an increased frequency of age-related diseases such as cancer. But its mechanism is unclear. TNFAIP8L2 (Tipe2) is an important negative regulator for homeostasis through inhibiting TLR and TCR signaling. Our work reveals that Tipe2 might have dual function by regulating senescence. One side, the overexpression of Tipe2 in CRC cells could induce typical senescent phenotype, especially exposure to oxidative stress. Tipe2 inhibits telomerase activity by regulating c-Myc and c-Est-2 binding to the hTERT promotor. Interestingly, *Tipe2* KO mice treated with D-Gal showed a less serious inverse of CD4:CD8 ratio, a lower percentage of Treg compared to WT. Besides, *Tipe2* KO mice were more tolerant to the initiation of AOM/DSS-induced CRC, accompanied by a lower level of Treg within IEL. Therefore, specific antibodies against CD25 effectively ameliorate tumorigenesis. These data suggest strongly that the overexpressed Tipe2 suppresses tumor cells proliferation and survival, but endogenous Tipe2 promotes the initiation of tumorigenesis when exposure to dangerous environment such as AOM/DSS-related inflammation.

## Introduction

Colorectal cancer (CRC) is the third leading cause of cancer death worldwide [1]. Studies demonstrated that inflammatory bowel diseases (IBD) including ulcerative colitis (UC) and Crohn’s disease were chronic inflammatory disorders of the gastrointestinal tract, strongly suggesting association with an increased risk of CRC development [2–4]. Recent studies showed that CRC might be related to cellular senescence [5, 6]. While the precise mechanism remains unknown.

Cellular senescence and telomere attrition are important processes which are involved in aging [7]. The decline of immune function is thought to be tightly associated with age-related diseases, such as cancer [8–10]. The hallmarks of immune senescence include an inverse of CD4/CD8 ratio, a shift from naïve to memory T cell phenotype, poor T-cell proliferative responses to stimuli, an increase of pro-inflammatory cytokines (such as IL-1, IL-6, TNF-α), these age-related changes result in the failure of homeostasis [11–14]. On the other hand, replicative senescence can be triggered by telomere shortening which is repaired and maintained by the activity of telomerase [15, 16]. However, the senescent cells induced by both replicative and cellular senescence are characterized by an enlarged and flattened shape, and generate specific biomarkers, such as an irreversible cell cycle arrest in the G_0_/G_1_ phase, increased content of β-galactosidase, overexpression of P21 and P16, etc. [17–19]. Substantial evidences have shown that blocking senescence accelerates [5] and induction of senescence inhibits CRC development [6]. Therefore, senescence might be a potential target for tumor therapy.

Tumor necrosis factor-α induced protein 8 like-2 (TNFAIP8L2, Tipe2) is the one member of the Tipe (TNFAIP8) family which can function as transfer proteins for phosphoinositide second messengers [20–22]. They are regulators for homeostasis, both in inflammation and carcinogenesis [22–24]. Their abnormal expressions are observed in various human diseases [21, 25–27]. *Tipe2*-deficient cells are hypersensitive to stimuli and defective in polarization and chemotaxis [20, 21] and *Tipe2*-deficient mice are resistant to leukocyte mediated inflammation [27]. In addition to negatively regulating pathogen-induced immune response, Tipe2 in DCs is also capable of promoting immune response under homeostatic conditions through the suppression of peripheral tolerance [25]. However, it is not clear if Tipe2 plays any role in senescence.

In the current study, we investigated the roles of Tipe2 in senescence using a colitis-associated CRC model and D-Gal-induced aging model. The results demonstrated that Tipe2 might play dual function in CRC by inducing senescence: suppresses the proliferation and survival of tumor cells but accelerates the initiation of AOM/DSS-induced CRC.

## Results

### *Tipe2*-deficiency resists aging, while the overexpression in CRC cells promotes cellular senescence

**Fig. 1:**
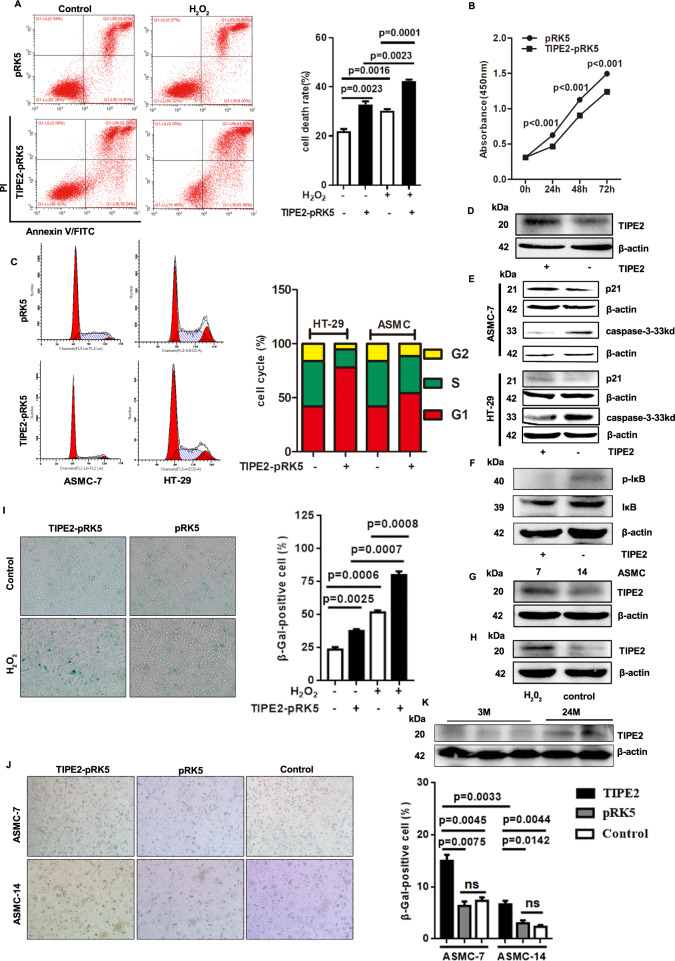
*Tipe2*-deficiency resists aging, while overexpression in cells promotes cellular senescence. The apoptotic cells were increased (**A**), while the cell proliferation (**B**) was inhibited in *Tipe2* transfected HT-29 cells. **C**, the cell cycle progression was inhibited in *Tipe2* transfected HT-29 or ASMC-7 cells. **D** Tipe2 expression in HT-29 cells. **E** caspase 3 (33kD) decreased, while P21 increased in Tipe2 overexpression HT-29 or ASMC-7 cells. **F** Tipe2 inhibits NF-κB signaling pathway. Tipe2 expression was higher in the 7^th^ than in 14^th^ passage AMSC cells (**G**). **H** H_2_O_2_ stimulation upregulated the Tipe2 expression in HT-29 cells. **I**, Sa-β-gal positive cells were increased in HT-29 cells transfected with Tipe2 plasmid with or without H_2_O_2_ stimulation. **J** Sa-β-gal positive cells were increased in primary cultured AMSC transfected with Tipe2 plasmid with or without H_2_O_2_ stimulation. **K** the levels of Tipe2 protein in WT mice of 24 months were significantly increased than that in 3 months ones. Data are representative of three independent experiments and expressed as means ± SEM. Significant difference between two groups was determined using an unpaired two-tailed Student’s *t*-test. *P < 0.05, **P < 0.01, ***P < 0.001. Data from **A** and **C** were collected using BECKMAN COULTER CytoFLEXS, then analyzed using CytExpert.

To determine the roles of Tipe2 in senescence, we detected sera biochemical parameters from C57BL/6 (WT) and *TIPE2*^*-/-*^ mice of different ages. The results showed that although the sera levels of albumin (ALB) decreased markedly both in WT and *Tipe2*-deficient mice with aging, the levels were significantly higher in *Tipe2*^*-/-*^ mice of 12 m than that in matched WT (Fig. S1A). The total plasma levels of alkaline phosphatase (ALP, Fig. S1B), alanine aminotransferase (ALT, Fig. S1C), glutamic oxaloacetylase (ASTL, Fig. S1D), cholesterol (CHO, Fig. S1E), and GGT (Fig. S1F) were upregulated significantly both in WT and *Tipe2*-deficient mice with aging, but were much lower in *Tipe2*-deficient mice (Fig. S1). Importantly, the levels of Tipe2 protein were much higher in WT mice of 24 m than in 3 m ones (Fig. 1K). These data suggested that Tipe2 might be associated with aging in mice.

Since senescent cells have typical features, such as an ability of lower proliferation and cell cycle arrest, we transfected Tipe2 plasmid to CRC cells and primary cells to determine the roles of Tipe2 in cell senescence. As shown in Fig. 1, Tipe2 can promote cell death and inhibit cell proliferation. The overexpression of Tipe2 in HT-29 cells significantly induced cell apoptosis, especially exposure to ROS stimuli (Fig. 1A and D). The cell growth was markedly suppressed (Fig. 1B) resulting from an inhibition of NF-κB signaling (Fig. 1F) [20], therefore the cell cycle arrested at the G_0_/G_1_ phase both in HT-29 and primary cultured 7^th^ ASMC cells transfected with Tipe2 plasmid (Fig. 1C). This phenomenon was confirmed by the increased expression of the P21 protein, and downregulation of the pro-caspase 3 (33 kd, Fig. 1E) both in CRC cells and primary cells. The upregulation of P21 is considered as an important marker for cellular senescence [28], suggesting that Tipe2 might promote cellular senescence.

Increased level of reactive oxygen species (ROS) generation is closely associated with cellular senescence [29]. Therefore, ROS (H_2_O_2_) stimulation and senescence-associated β-galactosidase (SA-β-Gal) staining were used to confirm the effect of Tipe2 on cellular senescence. Normally, the level of Tipe2 protein was much higher in the 7^th^ passage AMSC cells than in that 14^th^ cells (Fig. 1G). Tipe2 expression was upregulated significantly when cells exposure to H_2_O_2_ stimulation (Fig. 1H). The ratio of SA-β-Gal staining positive cells in pRK5-Tipe2 transfected cells was much higher than that in pRK5 controls (Fig. 1I), especially exposure to H_2_O_2_ stimulation, suggesting that ROS stimuli markedly increased the number of senescent cells induced by Tipe2 overexpression (Fig. 1I). The same phenomena were observed in primary cultured cells (Fig. 1J). These data indicated that Tipe2 might promote cellular senescence, especially exposure to oxidative stress.

### Tipe2 promotes replicative senescence by regulating telomerase activity

**Fig. 2:**
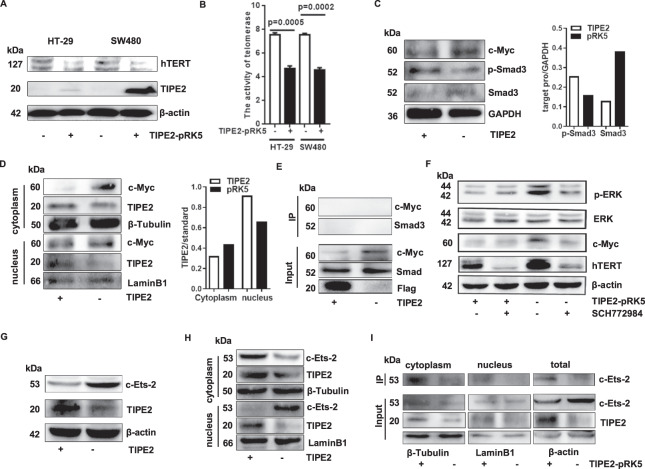
Tipe2 inhibits telomerase activity through regulating c-Myc and c-Ets-2. **A** the overexpression of Tipe2 in HT-29 and SW480 cells inhibited the expression of hTERT. **B** the telomerase activity was significantly decreased in Tipe2 transfected HT-29 and SW480 cells. **C** Tipe2 overexpression in HT-29 cells downregulated c-Myc protein while upregulated the phosphorylation level of p-Smad3. Tipe2 downregulated the cytoplasm c-Myc but not nucleus c-Myc (**D**), and didn’t bind to c-Myc (**E**). **F** Tipe2 inhibited the phosphorylation of ERK and the expression of c-Myc. Tipe2 inhibited c-Ets-2 expression (**G**), bound to cytoplasmic c-Ets-2 (**I**) resulting a decreased level of the nucleus c-Ets-2 (**H**) in HT-29 cells. The images were analyzed using ImageJ software.

Replicative senescence can be triggered by the shortening of chromosome ends (also called telomeres which are regulated by telomerase activity). To explore the role of Tipe2 on telomere, the telomerase activity was determined in Tipe2 overexpressed cells. The results showed that the overexpression of Tipe2 in HT-29 and SW480 cells could inhibit the expression of hTERT (Fig. 2A). Accordingly, the telomerase activity decreased significantly (Fig. 2B). c-Myc is an important regulator for telomerase activity [30]. We found that the overexpression of Tipe2 in tumor cells markedly decreased the protein level of c-Myc, but increased the phosphorylation level of p-Smad3 (Fig. 2C). Further studies showed that Tipe2 downregulated the cytoplasm c-Myc, but did not affect nucleus c-Myc (Fig. 2D). Very interestingly, the level of nucleus Tipe2 was significantly higher compared with control cells (Fig. 2D). While IP results showed that Tipe2 couldn’t bind to c-Myc (Fig. 2E), suggesting that Tipe2 downregulated the expression of c-Myc, but did not affect its nucleus translocation. Figure 2F showed that the effect of Tipe2 on c-Myc downregulation might be associated with the inhibition of the phosphorylation of ERK. But the inhibitor SGH772984 for ERK didn’t suppress the effect of Tipe2 on hTERT, suggesting that might have other factors regulate hTERT activity through Tipe2.

c-Ets-2, a genuine cancer-specific transcription factor, upregulates the telomerase activity by binding to the promoter of hTERT [31]. As expected, the overexpression of Tipe2 markedly downregulated the c-Ets-2 expression (Fig. 2G). Interestingly, Tipe2 did not affect cytoplasm c-Ets-2, but decreased its nucleus level (Fig. 2H). Further IP experiment showed that Tipe2 blocked c-Ets-2 translocation from cytoplasm to nucleus by binding to c-Ets-2 in the cytoplasm (Fig. 2I, Fig. S2), suggesting that Tipe2 might suppress telomerase activity by blocking the translocation of c-Ets-2.

### *Tipe2*-deficiency is resistance to aging in the D-gal-induced mice

**Fig. 3:**
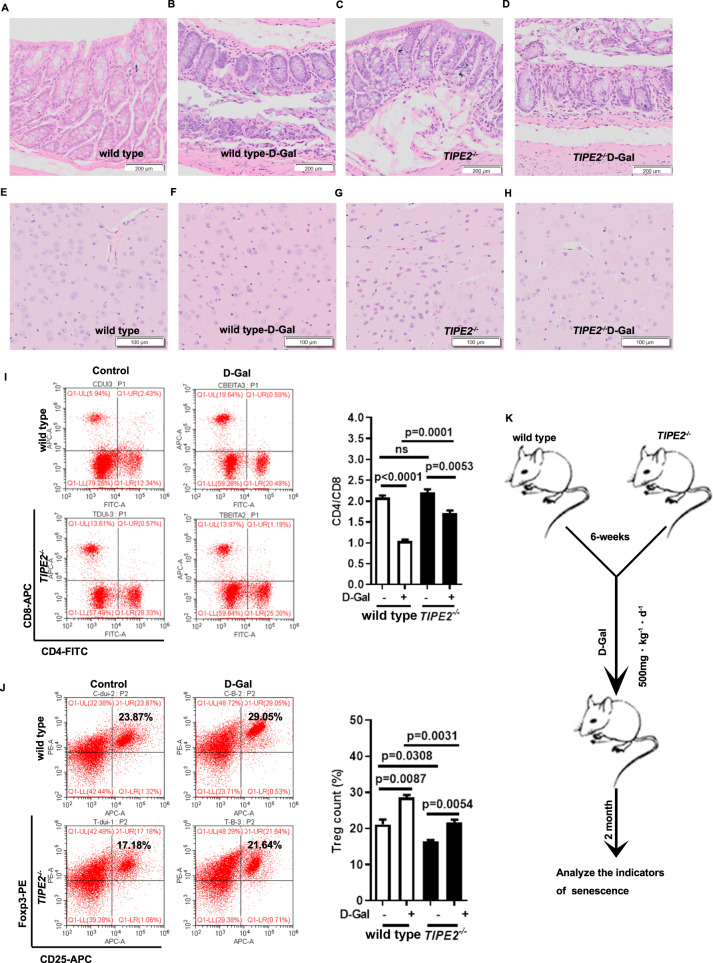
*Tipe2* KO mice were less susceptible to D-Gal-induced aging. The HE staining of intestinal tissues (**A**–**D**) and brain (**E**–**H**) from D-Gal induced mice models. Without D-Gal the intestinal or brain from WT (**A**, **E**) and *Tipe2*^-/-^ (**C**, **G**) showed no difference. Severe age-related morphology was observed in intestinal and brain from WT mice (**B**, **F**) treated with D-Gal compared to *Tipe2*-deficient mice (**D**, **H**). *Tipe2* KO mice treated with D-Gal showed a less serious inverse of CD4:8 ratio (**I**) and a lower percentage of Tregs (**J**) compared to WT controls. **K** The procedure of D-Gal induced aging mouse model. Data are representative of three independent experiments and expressed as means ± SEM. Significant difference between two groups was determined using an unpaired two-tailed Student’s *t*-test. *P < 0.05, **P < 0.01, ***P < 0.001. Data from I and J were collected using BECKMAN COULTER CytoFLEXS, then analyzed using CytExpert.

D-Galactose (D-Gal), a reducing sugar normally present in the body, is widely used to induce an ideal model to study the possible mechanism of aging-related disease [32]. To address the role of Tipe2 in aging, *Tipe2*^*-/-*^ mice and WT controls were administrated with D-Gal to induce aging models (Fig. 3K). WT mice treated with D-Gal showed more severe age-related losses in villous and enterocyte heights, a decreasing number of goblet cells, and the depth of crypt in the intestinal morphology (Fig. 3B and D). While there was no significant difference between WT and *Tipe2*^*-/-*^ mice without D-Gal treatment (Fig. 3A and C). Besides, the microglial cells were reduced in length, showed less branching and vacuole formation in WT mice treated with D-Gal compared to matched *Tipe2*^*-/-*^ mice, especially neuron cells showed more serious apoptotic or aging-related morphology including chromatic agglutination, karyopyknosis, nuclear fragmentation, and cytoplasmic vacuolation (Fig. 3F and H). But there was no significant difference between WT and *Tipe*2-deficient mice without D-Gal (Fig. 3E and G).

Oxidative stress plays an important role in inducing brain aging. Superoxide dismutase (SOD), an important enzyme involved in the removal of ROS, and monoamine oxidase (MAO, a brain regional mitochondrial enzyme), malondialdehyde (MDA, an end-product of ROS-induced peroxidation), are widely used as the oxidative stress biomarkers [33]. In this study, D-Gal could downregulate the level of SOD in serum both in WT and *Tipe2*^*-/-*^ mice, but the SOD level from *Tipe2*^*-/-*^ mice treated with D-Gal was much higher (Fig. S3A). On the other hand, the levels of MDA (Fig. S3B) and MAO (Fig. S3C) were significant higher in D-Gal treated both WT and *Tipe2* KO mice, while the latter were much lower, suggesting that *Tipe2*-deficient mice might resist D-Gal induced aging through resisting oxidative stress.

The inverted CD4:8 ratio and the upregulation of Tregs are considered as aging-associated immune markers [34]. In this study, we found that *Tipe2* KO mice treated with D-Gal showed a less serious inverse of CD4:8 ratio. Although there was no significant difference about the baseline ratio, D-Gal could significantly downregulate the ratio both in *Tipe2*^*-/-*^ mice and WT controls, but it was significantly higher in D-Gal-treated *Tipe2*^*-/-*^ mice (Fig. 3I). Furthermore, the percentage of Tregs in *Tipe2*^*-/-*^ mice was significantly lower than that in WT without D-Gal treatment (Fig. 3J). D-Gal could upregulate Tregs levels both in *Tipe2*^*-/-*^ mice and matched WT, but the level was much lower in *Tipe2*^*-/-*^ mice (Fig. 3J). These results suggested that *Tipe2*-deficiency might resist immune senescence in D-Gal-induced mice.

### *Tipe2*-deficiency in naïve CD4^+^ T cells might inhibit iTreg differentiation

**Fig. 4:**
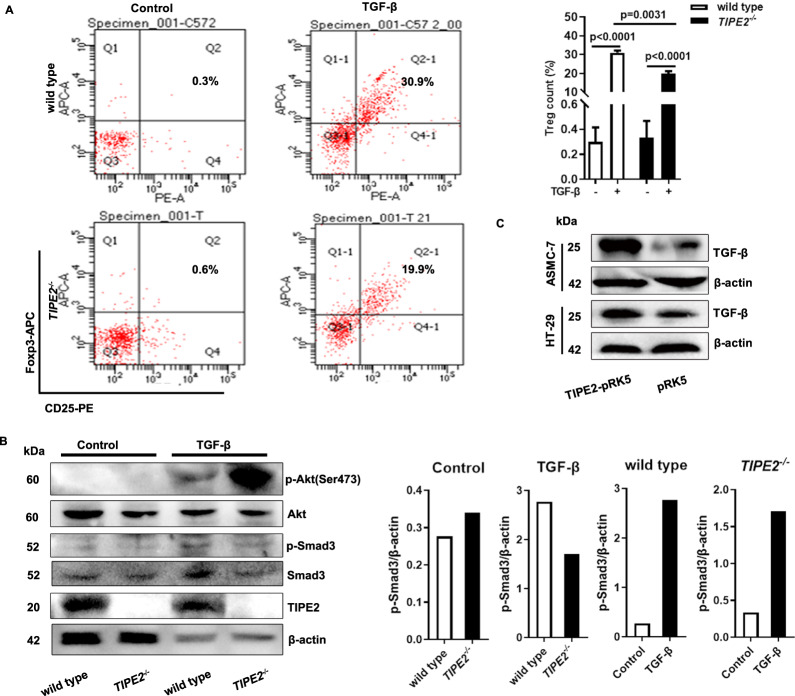
*Tipe2*-deficiency in naïve CD4^+^ T cells inhibits iTreg differentiation. **A** Showed a lower percentage of iTregs differentiated from *Tipe2*-deficent naïve CD4^+^ T cells in vitro. Data were collected using BECKMAN COULTER CytoFLEXS, then analyzed using CytExpert. **B** Showed an increased level of p-Akt and decreased p-Smad3 in *Tipe2*-deficent naïve CD4^+^ T cells with TGF-β stimulation. The images were analyzed using ImageJ software. **C** The overexpression of Tipe2 upregulated TGF-β expression in HT-29 or AMSC cells. Data are representative of three independent experiments and expressed as means ± SEM. Significant difference between two groups was determined using an unpaired two-tailed Student’s *t*-test. *P < 0.05, **P < 0.01, ***P < 0.001.

We found that the levels of Tregs from *Tipe2*^*-/-*^ mice treated with D-Gal were significantly lower compared with WT/D-Gal mice (Fig. 3J). Liu R et al. reported that Tipe2 in DC could inhibit the induction of pTregs in the gut mucosa [35]. To verify its role in Treg differentiation, naïve CD4^+^ T cells separated from *Tipe2*^*-/-*^ or C57 mice were cultured with TGF-β to induce Tregs (iTregs) [36]. The results showed that TGF-β upregulated the levels of iTreg markedly both in *Tipe2*^*-/-*^ and *Tipe2*^*+/+*^ naïve CD4^+^ T (Fig. 4A), but it was much lower in *Tipe2*^*-/-*^ ones (Fig. 4A). Results from WB revealed that *Tipe2*-deficiency could increase the level of p-Akt phosphorylation in TGF-β activated naïve CD4^+^ T cells, while decrease the phosphorylation of p-Smad3 (Fig. 4B). The activation of TGF-β/Smad signaling pathway is associated with the increase in senescent phenotype [37]. Therefore, to confirm our results, TGF-β/Smad3 signaling pathway was detected in primary and tumor cell lines transfected with or without Tipe2 plasmid. We found that the overexpression of Tipe2 upregulated the expression of TGF-β protein both in primary cultured 7^th^ ASMC cells and CRC cells (Fig. 4C), accompanied by the upregulation of p-Smad3 (Fig. 2C). The activation of the Smad3 pathway and the inhibition of Akt-signaling are considered the most important signaling pathways during iTreg differentiation [38]. These data indicated that Tipe2 might enhance senescence through promoting the naïve CD4^+^ T cells to differentiate into iTregs with TGF-β stimuli.

### *Tipe2-*deficient mice are higher resistance to AOM/DSS-induced tumorigenesis

**Fig. 5:**
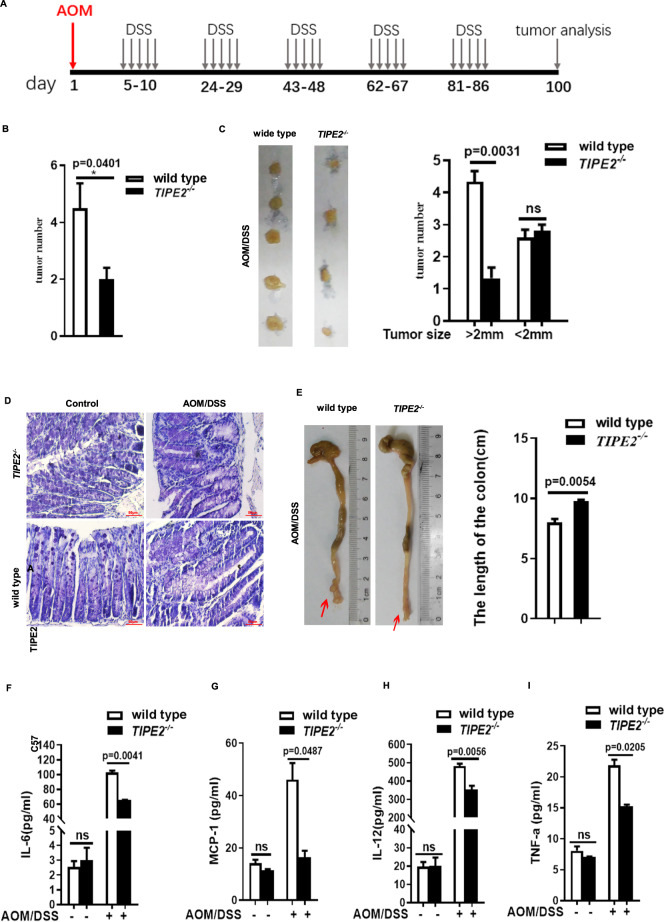
*Tiep2*-deficient mice were more resistant to AOM/DSS-induced tumorigenesis. **A** The protocol of AOM/DSS-induced CRC. The tumor number was significant lower in *Tipe2*-deficent AOM/DSS models (**B**), especially tumors less than 2 mm in diameter (**C**). Tipe2-defiency showed less severe intestinal morphology (**D**) accompanied by longer length of the colon (**E**) in AOM/DSS-induced mice. *Tipe2*-deficent AOM/DSS models showed lower serum levels of proinflammatory cytokines, such as IL-6 (**F**), MCP-1 (**G**), IL-12 (**H**) and TNF-α (**I**). Data are representative of three independent experiments and expressed as means ± SEM. Significant difference between two groups was determined using an unpaired two-tailed Student’s *t*-test. *P < 0.05, **P < 0.01, ***P < 0.001.

AOM is a potential DNA damage-inducing agent that is commonly used to induce colorectal cancer (CRC). To investigate the role of Tipe2 in colitis-associated CRC, we used a protocol that combines the AOM carcinogen with DSS-induced colitis (Fig. 5A) [39]. We found that the total tumor number of AOM/DSS-induced CRC from *Tipe2*^*+/+*^ mice was higher than that from *Tipe2*^*-/-*^ (Fig. 5B), especially in tumors with greater diameter more than 2 mm (Fig. 5C), suggesting that the occurrence of AOM/DSS-induced CRC in *Tipe2*^*+/+*^ mice was probably earlier than that in *Tipe2*^*-/-*^ mice. The *Tipe2*^*+/+*^ mice showed more severe inflammation with many areas of crypt lost (Fig. 5D). They presented widespread mucous glands destruction and derangement, epithelial atypia proliferation, while *Tipe2* knockout could alleviate cancer atypia and inflammation. The length of the colon from *Tipe2*^*+/+*^ mice was significantly shorter than *Tipe2*^*-/-*^ ones (Fig. 5E). Inflammatory mediators are upregulated during AOM/DSS-induced CRC, but *Tipe2*^*-/-*^ mice showed lower serum levels of inflammatory cytokines, such as IL-6 (Fig. 5F), MCP-1 (Fig. 5G), IL-12 (Fig. 5H), and TNF-α (Fig. 5I). The changes of these cytokines, especially IL-6, are consistent with the notion that IL-6 is critical for colon tumor development. These data suggested that Tipe2 might promote the initiation of AOM/DSS-induced CRC.

### *Tipe2*-deficiency was more susceptible to anti-CD25-induced Treg depletion

**Fig. 6:**
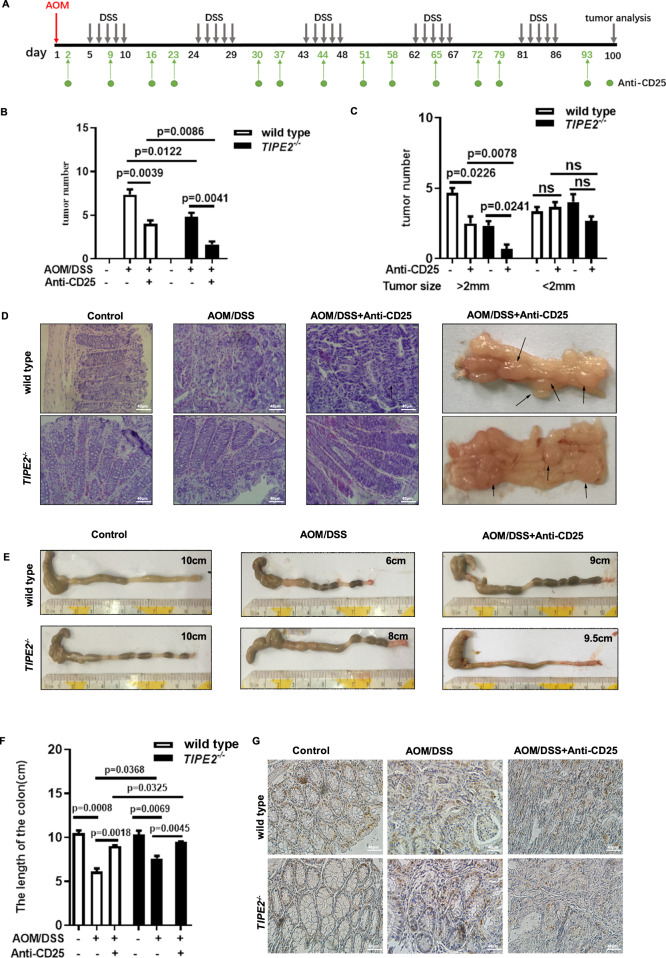
*Tipe2*-deficiency was susceptible to anti-CD25-induced Treg depletion. **A** The used protocol in the experiment. The total tumor number (**B**) or tumors less than 2 mm in diameter (**C**) was significant lower in *Tipe2*-deficent AOM/DSS models, especially in models with anti-CD25 treatment. Tipe2-defiency showed less severe intestinal inflammation (**D**) accompanied by longer length of the colon (**E** and **F**) in AOM/DSS models, especially with anti-CD25 treatment. **G** The BrdU positive cells decreased in Tipe2-deficent AOM/DSS models, especially with anti-CD25 treatment. Significant difference between two groups was determined using an unpaired two-tailed Student’s *t*-test. *P < 0.05, **P < 0.01, ***P < 0.001.

To confirm the role of Tipe2 on iTreg differentiation, anti-CD25 mAb was used to block the effect of Treg in AOM/DSS-induced colon cancer. The used protocol was shown in Fig. 6A. As expected, the total CRC number from AOM/DSS/anti-CD25 treated mice were much lower than that from the mice without anti-CD25 injection (Fig. 6B), especially tumors with size more than 2 mm (Fig. 6C, left panel). Very interestingly, the tumor number from anti-CD25 treated *Tipe2*^*-/-*^ mice was significantly lower compared with anti-CD25 treated *Tipe2*^*+/+*^ controls (Fig. 6B), especially tumors with size more than 2 mm (Fig. 6C, left panel). There was no significant difference in tumor number with size less than 2 mm between *Tipe2*^*+/+*^ and *Tipe2*^*-/-*^ mice with or without anti-CD25 treatment (Fig. 6C, right panel). Tissues from antibody administrated *Tipe2*^*+/+*^ mice showed more serious inflammatory morphology compared with matched *Tipe2*^*-/-*^ mice, but the severity of the inflammation and injury of AOM/DSS induced CRC from two groups was ameliorated effectively after anti-CD25 injection (Fig. 6D, E and F). BrdU labeling showed more positive cells (tumor cells) in AOM/DSS treated WT compared to matched *Tipe2* KO mice, but markedly decreased in anti-CD25 injected mice (Fig. 6G). WB results showed that the levels of c-Myc, activation of ERK and NF-κB in AOM/DSS-treated WT models were much higher than that in *Tipe2*-deficient models (Fig. S4).

**Fig. 7:**
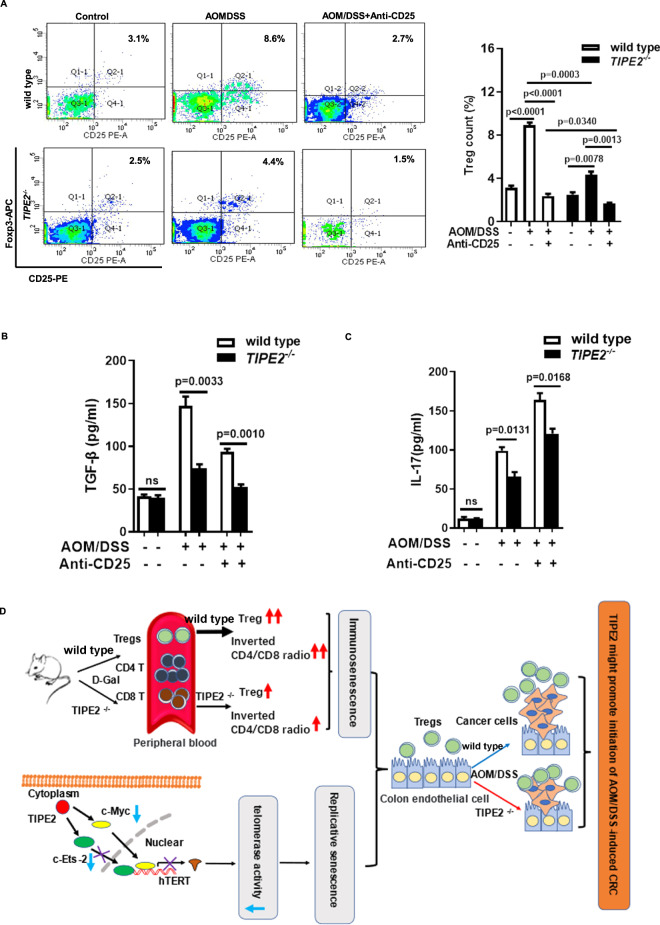
Cytokines detection and probable mechanism. **A** The percentage of Tregs from IEL separated from *Tipe2*-defienct AOM/DSS models was significant lower than WT controls, especially with anti-CD25 treatment. Data were collected using BECKMAN COULTER CytoFLEXS, then analyzed using CytExpert. *Tipe2*-deficent AOM/DSS models showed lower serum levels of TGF-β (**B**) and IL-17 (**C**) with or without anti-CD25 antibody treatment. **D** Tipe2 inhibits tumor cell growth while promotes the initiation of AOM/DSS-induced colon cancer through regulating senescence.

Results of flow cytometry analysis showed that AOM/DSS treatment upregulated the percentage of CD25^+^FoxP3^+^ T cell (Treg) in IEL separated from both *Tipe2*^*-/-*^ mice and *Tipe2*^*+/+*^ (Fig. 7A), but was significantly lower in AOM/DSS-induced *Tipe2*^*-/-*^ mice (Fig. 7A). With anti-CD25 treatment, the percentage of Treg decreased significantly in both two groups (Fig. 7A). Very importantly, the anti-CD25-treated *Tipe2*^*-/-*^ mice showed a much lower Treg ratio (Fig. 7A) and a decrease in the serum level of TGF-β (Fig. 7B), but did not affect the levels of IL-17 (Fig. 7C). These data suggested that the anti-CD25 might ameliorate AOM/DSS-associated CRC via blocking CD25^+^Foxp3^+^ Treg differentiation, especially in *Tipe2*-deficient mice.

## Discussion

Our work reveals that Tipe2 might have dual function by regulating senescence: overexpression in tumor cells inhibits tumor cell proliferation and survival, while endogenous *Tipe2*-deficiency suppresses colitis-associated colorectal cancer (CRC) initiation. One side, the overexpression of Tipe2 in CRC cells suppresses cell growth, promotes cell cycle arrest, inhibits telomerase activity by regulating transcription factors, such as c-Myc and c-Est-2, which bind to the hTERT promotor. The percentage of SA-β-Gal staining positive cells (senescent cells) is increased in Tipe2 overexpression cells, especially exposure to oxidative stress. This is accompanied by a less serious inverse of CD4: CD8 ratio, a lower percentage of Treg in PBMC from *Tipe2* KO mice treated with D-Gal compared to matched WT. Besides, *Tipe2*-deficiency is more tolerant to the initiation of AOM/DSS-induced CRC. This is accompanied by a lower level of Treg within IEL from the AOM/DSS-treated *Tipe2* KO mice. Therefore, specific antibodies against CD25 effectively ameliorate tumorigenesis. These data suggest strongly that through inducing senescence, Tipe2 suppresses tumor cells proliferation and survival, but promotes the initiation of tumorigenesis when exposure to dangerous environment such as AOM/DSS-related inflammation.

In agreement with large amounts of previous evidence for the suppressor role of Tipe2 in tumor cells [21–23], we demonstrate that the overexpression in CRC cells can inhibit cell growth, upregulates P21 expression and promotes cell cycle arrest in G_0_/G_1_ phase, which are considered to be senescent phenotype. The phenomenon might result from that Tipe2 involved in replicative senescence by regulating hTERT. Replicative senescence can be triggered by the shortening of chromosome ends (also called telomeres, which are regulated by telomerase activity). hTERT is the main catalytic unit for telomerase activity. We found that the overexpression of Tipe2 markedly downregulated the expression of c-Myc and c-Ets-2, which are important regulators for hTERT transcription [30, 31]. Tipe2 also blocks the nucleus translocation of c-Ets-2. Accordingly, the telomerase activity decreased significantly. Cells with Tipe2 overexpression are inclined to senescent phenotype. These data further confirm the notion that the overexpression of Tipe2 plays suppressor role in tumor cells [20–22].

However, the existence of Tipe2 might be favorable to the initiation of AOM/DSS induced CRC. Present papers published on Tipe2 and colon cancer suggest Tipe2 a tumor suppressor but very few experiments to verify [23, 40, 41], especially no AOM/DSS CRC model. The AOM/DSS protocol is highly dependent on DSS which induces epithelial inflammation and apoptosis. Consistent with Lou’s report that *Tipe2*-deficiency suppressed the DSS-associated colitis in mouse model [42], we reported that AOM/DSS-treated *Tipe2* KO mice exhibited significantly less severe colitis and this might result in less severe tumorigenesis. This was accompanied by a lower percentage of CD4^+^CD25^+^Foxp3^+^ Treg cells within IEL, suggesting that Tipe2 might be associated with Treg differentiation.

CD4^+^CD25^+^ Tregs are instrumental in the maintenance of immunological tolerance. Several reports revealed that Tipe2 was associated with the suppressive function of tTregs [43, 44]. It promoted the thymus egress of tTregs and did not affect tTregs development [45]. But its expression in DC could inhibit the induction of pTregs in the gut mucosa [35]. Therefore, to confirm the role of Tipe2 in Treg function and development, the naïve peripheral CD4^+^CD25^-^ T cells were separated from *Tipe2* KO mice and WT. These cells were induced to differentiate into CD4^+^CD25^+^Foxp3^+^ Tregs (iTreg) through co-stimulation with TCR and TGF-β [36]. We found that *Tipe2*-deficient naïve CD4^+^ T cells were less susceptible to differentiate into iTregs through regulating TGF-β/Smad signaling pathway in vitro, but more susceptible to the Tregs depletion induced by anti-CD25 antibody in vivo.

The suppressive mechanisms of Treg on immune response might be associated with Treg-induced effector T-cell senescence [46]. We found that D-Gal-treated *Tipe2*-deficient mice showed a less serious reverted CD4:8 ratio accompanied by a downregulation of Treg percentage. Very interestingly, AOM/DSS-treated *Tipe2*-deficient mice showed less severe inflammation and tumorigenesis, especially exposure to anti-CD25 depletion. These data indicated that AOM/DSS-treated *Tipe2*-deficient mice showed less severe CRC might due to the suppression of iTreg differentiation induced by Tipe2 in naïve CD4^+^ T cells. Targeting Treg-induced effector T-cell senescence might be a checkpoint for immunotherapy against cancer and other age-related diseases.

*Tipe2*-deficient mice were less susceptible to D-Gal-induced aging. *Tipe2* KO mice showed less severe age-related intestinal and brain morphology and lower serum levels of oxidative stress biomarkers, such as MDA and MAO, while a higher level of SOD which plays important role in the removal of ROS. Very interestingly, a less serious inverted CD4:8 ratio and lower percentage of Tregs, which are considered as aging-related immune markers [34], were observed in D-Gal-treated *Tipe2*-deficient mice. These results suggested that *Tipe2*-deficiency might be resistant to D-Gal-induced senescence in mice.

Taken together, our work reveals a dual function of Tipe2 on AOM/DSS-induced CRC through promoting senescence. *Tipe2*-deficiency is less susceptible to senescence and might be a checkpoint for immunotherapy against cancer and other age-related diseases.

## Materials and methods

### Antibodies

The following antibodies or reagents used for flow cytometry were from Biolegend: FITC anti-mouse CD4 (Cat#100509), PE anti-mouse CD25 (Cat#102007), Alexa Fluor® 647 anti-mouse FOXP3 (Cat#126407), FITC anti-mouse CD3 (Cat#100203) or PerCP/Cy5.5 anti-mouse CD3ε (Cat#100327), APC anti-mouse CD8a (Cat#100711), TruStain fcX™ (anti-mouse CD16/32, Cat#101320) and True-Nuclear™ Transcription Factor Buffer Set (Cat#424401) was used for blocking non-specific binding of immunoglobulin to the Fc receptors and intracellular staining perm wash buffer, respectively. Antibodies to hTERT (Cat#ab230527, 1:1000 dilution) was from Abcam, ERK (Cat#4694 T, 1:1000 dilution), p-ERK (Cat#4376 T, 1:1000 dilution), Akt (Cat#2920 T, 1:1000 dilution), p-Akt (Ser473) (Cat#9271 S, 1:1000 dilution), p-Smad3 (Cat#9513 T) and Smad3 (Cat#9520 T, WB 1:1000 dilution; IF 1:50 dilution), c-Myc (Cat#9402 S, WB 1:1000 dilution; IF 1:50 dilution), c-Ets-2 (Cat#66476 S, WB 1:1000 dilution; IF 1:50 dilution), IκB (Cat#9242 T, 1:1000 dilution), p-IκB (Cat#9246 T, 1:1000 dilution), caspase-3 (Cat#9662 T, 1:1000 dilution), P21 (Cat#37543, 1:1000 dilution), were from Cell Signaling Technology (CST). Antibodies to β-actin (Cat#66009-1-Ig, 1:1000 dilution), β-tubulin (Cat#66240-1-Ig, 1:1000 dilution), LaminB1 (Cat#66095-1-Ig, 1:1000 dilution), and TGF-β (Cat#21898-1-AP, 1:1000 dilution) were from Proteintech, CD3ε and CD28 (Cat#114.52D/114.53D) were from Invitrogen.

### AOM/DSS colitis-associated carcinoma mouse model

The *Tipe2*-deficient (*Tipe2*^*−/−*^) mice (C57BL/6 J background) have been described previously [20]. C57BL/6 J mice (WT, *Tipe2*^*+/+*^) were purchased from the Shanghai Laboratory Animal Center of the Chinese Academy of Science. All mice used were male and 6-7 weeks old, randomly divided into groups and no blinding was used. Mice were housed under pathogen-free conditions of humidity (50 ± 10%), lighting (12 h light/dark cycle), and temperature (25 ± 2 °C) with pure water and a freely accessible pelleted basal diet in the University of Shandong Animal Care Facilities. All handlings and procedures were carried out as per the protocol approved by the Ethical Committee of the School of Basic Medical Science, Shandong University.

For colitis-associated carcinoma models, pilot studies were used for estimation of the sample size required to ensure adequate power. *Tipe2*-deficient mice and WT controls were randomly divided into four groups (five per experimental group): *Tipe2* KO treated with AOM/DSS or saline control, WT treated with AOM/DSS or saline control. In the first week the test groups were injected intraperitoneally with a single-dose of azoxymethane (AOM, Cat#A5486, Sigma-Aldrich, St. Louis, MO, 10 mg/kg). After 5 days, dextran sodium sulfate (DSS, 2%, Sigma-Aldrich, Cat#160110) was dissolved in drinking water and administrated to mice for 5 days, followed by 14 days of regular drinking water. The protocol was described in Fig. 5A. The same protocol was performed with intraperitoneal normal saline and drinking distilled water instead of the AOM/DSS treatment in the control groups. For Treg depletion model, *Tipe2* KO mice and WT were randomly divided into six groups (six per experimental group): *Tipe2* KO treated with AOM/DSS/anti-CD25 antibody, AOM/DSS or saline control, WT treated with AOM/DSS/anti-CD25 antibody, AOM/DSS or saline control. The depletion model mice were treated with anti-CD25 antibodies (Sungenebiotec, China) as described in Fig. 6A. The body weight and health conditions of the mice were recorded every week. On the last night before mice were sacrificed, animals and their respective controls were injected intraperitoneally with BrdU (5-bromo-2′-deoxyuridine, 10 mg/kg, Sigma-Aldrich, Cat#19-160) to label newly born cells. Animals were sacrificed 100 days after the AOM injection. Colons were excised and flushed with cold PBS, size measurements were performed using a digital caliper in a blinded fashion. Then the colons were fixed in 10% formalin solution (Sigma) at room temperature, and paraffin-embedded for HE staining. All data were expressed as mean ± SEM. Differences between two groups were determined as unpaired two-tailed Student’ s *t*-test. *P* < 0.05 was considered as statistically significant.

### Aging animal model

Male WT and *Tipe2* ^*-/-*^ mice were sacrificed at the age of 3 (*n* = 3) and 24 months (*n* = 3). Fresh sera were collected to determine albumin (ALB), alkaline phosphatase (ALP), alanine aminotransferase (ALT), aspartate aminotransferase (ASTL), gamma glutamyl transpeptidase (GGT), and total cholesterol (CHO) using commercial kits (Changchun huili, C061/C063; Jiubang, CK-E28753) according to the manufacturer’s instructions. One part of the tissues such as spleen was frozen in −80 °C to detect Tipe2 protein using western blot.

Senescent *Tipe2*-deficient (*n* = 6) and WT (*n* = 6) mice were induced by single-dose intraperitoneal injection of D-Galactose (D-Gal, 500 mg/kg, Sigma-Aldrich, Cat#G0750) every day for 2 months and were sacrificed in the 14^th^ week, and the brain, large bowels were excised for evaluating histological evidence. Fresh sera were collected to determine Superoxide dismutase (SOD) (Cat#BC0170, Solarbio, Beijing, China), monoamine oxidase (MAO) (Cat#K009679P, Solarbio), Malondialdehyde (MDA) (Cat#BC0025, Solarbio). Peripheral blood was collected to detect CD4^+^ T cells, CD8^+^ T cells, and Tregs by Flow cytometry.

### Hematoxylin–eosin (HE) staining and histological analysis

The formalin-fixed colon tissues were embedded in paraffin blocks. Sliced sections about 4 mm were deparaffinized and rehydrated by a xylene–ethanol–water gradient system. Hematoxylin and eosin (HE) staining was performed followed by a dehydrating process. Histopathological examination was performed under a light microscope by Olympus (Waltham, MA, USA). Neoplasms and inflammations were analyzed and diagnosed as the established criteria. Histopathological evaluation was determined by two pathologists from the pathology department of Qilu Hospital (Dr. Chao Ma and Dr. Chunyan Hao, Shandong, China) who were not aware of the experimental protocols.

### Detection of inflammatory cytokines

ELISA kit was used to detect the expression of several inflammatory cytokines such as MCP-1 (Cat#432704, Biolegend), IL-17A (Cat#433007, Biolegend), TGF-β (Cat#432507, Biolegend), IL-6 (Cat#1210602, DAKEWE), IL-12p70 (Cat#1211202, DAKEWE) and TNFα (Cat#1217202, DAKEWE) in the sera of mice models. All analyses and calibrations were performed in duplicate. Optical densities were determined using an absorbance microplate reader (Tecan, Infinite M200, Switzerland) at 450 nm. Graph prism 8 Data Analysis software was used to analyze all data. Differences between two groups were determined as unpaired two-tailed Student’ s *t*-test.

### The separation of Tregs from IEL

According to the procedure described in [47], the Tregs were separated from IEL. The colon was removed from the mouse model and washed in ice-cold RPMI (HyClone, USA). IEL was isolated and transfered to a clean tube. Flow cytometry was performed to analyze Tregs from IEL.

### iTreg differentiation

6–8 weeks male *Tipe2* KO and WT mice (five per experimental group) were sacrificed and spleens were collected into sterile complete RPMI. Single cell suspensions were obtained by mashing the spleen and passing cells through 70 μm cell strainer (BD, Franklin Lakes NJ, USA). Naïve CD4^+^ T cells were obtained according to the protocol of Miltenyi. 24 well sterile tissue culture plates (Corning, NY, USA) were coated with 4 μg/mL anti-CD3ε and 4 μg/mL anti-CD28 in 0.5 mL/well PBS at 37 °C for 2 h. Purified naïve CD4^+^ T cells which was isolated from *Tipe2*^*-/-*^ or WT spleen were washed and resuspended in complete RPMI media supplemented with 100 IU/mL rhIL-2 (Peprotech, Cat#AF-200-02-10, Rocky Hill, NJ, USA) and 5 ng/mL rhTGF-β (Peprotech, Cat#100-21C-2). 3 × 10^5^ cells were added to the wells at a volume of 1 mL/well. Cells were cultured at 37 °C 5% CO_2_ for 3 days and harvested for flow cytometry.

### Flow cytometry

Surface markers were stained in PBS with antibodies for 20 min at room temperature, the cells were fixed in Cytoperm/Cytofix (Cat#88-8824-00, Invitrogen, USA), permeabilized with Perm/Wash Buffer (Invitrogen) and stained with Biolegend conjugated antibodies. iTreg stained with FITC-anti-CD4, PE-anti-CD25 and APC-anti-Foxp3a. Cell cycle and apoptosis were determined in HT-29 and ASMC cells transfected with Tipe2-pRK5 or vector control. Data were collected using BECKMAN COULTER CytoFLEXS, then analyzed using CytExpert.

### Cell culture

Colon cancer cell line (HT-29, TCHu103) obtained from National Collection of Authenticated Cell Culture (NCACC) and primary lung airway smooth muscle cells in mice (ASMC, CP-M005) obtained from Procell Life Science & Technology were cultured in the RIPA1640 (HyClone) medium containing 10% FBS (HyClone). SW480 (TCHu172) obtained from NCACC was cultured in L-15 medium containing 10% FBS. The cells were incubated in wet cell incubator with 5% CO_2_ at 37 °C. The expression of Tipe2 was detected using western blot in the 7^th^ generation and 14^th^ generation ASMC cells. Tipe2-pRK5 plasmid was transfected into ASMC or HT-29 cells treated with or without H_2_O_2_ (Sigma-Aldrich, Cat#88597). SA-β-Gal (Byeotime, Cat#C0602, Shanghai, China) staining was used to detect cell senescence. The relationship between the levels of Tipe2 protein and cell senescence was analyzed.

### Proliferation analysis

The cleaved protein of caspase-3 (CST, USA) and P21 (CST) were detected using western blot, the cells proliferation was detected using CCK-8 kit (APExBIO, Cat#K1018, USA). About 1 × 10^4^ HT-29 cells transfected with pRK5-Tipe2 or vector control were cultured in a 96-well plate. When reaching 90% confluence, cells were treated with or without H_2_O_2_ for different times. After treatment with 10 μL of CCK-8 reagent for 2 h, absorbance was determined at 450 nm. The experiments were performed with six replicated wells per sample, and the assays were conducted in triplicate.

### Telomerase activity analysis

The activity of telomerase was detected by TeloTAGGG Telomerase PCR ELISA Kit (Roche Diagnostics GMBH Mannheim, Cat#11854666910). The relationship between the levels of Tipe2 protein and hTERT activity is analyzed.

### Cytoplasmic and nuclear protein extraction

Cell cytoplasm and nucleus protein were collected with NE-PER Nuclear and Cytoplasmic Extraction Reagents (Pierce) according to the manufactures’ protocol. The distributions of protein in cytosol and nucleus were analyzed by western blot.

### Co-immunoprecipitation assay

After the cultured cells washed with pre-chilled PBS for 2 times, these cells were added in cold RIPA lysis buffer. After centrifuge, the supernatant (cell extract) was transferred to a new tube for IP assay. Firstly, about 100 μg cell extract was pre-cleared with protein A agarose (Sigma-Aldrich, Cat#IP02), then incubated with anti-Tipe2 (1:200, JinSiTe, Nanjing, China) antibody at 4 °C overnight with constant rotation. Then, the protein/antibody mixture was incubated with the pretreated Protein A-sepharose beads (Sigma-Aldrich). After the precipitant was collected and washed, the beads were resuspended in SDS-PAGE loading buffer and heated at 95 °C for 5 min. The supernatant was analyzed by SDS-PAGE and western blotting.

### Western blot

50 μg protein was separated by 12% SDS-PAGE and transferred to polyvinylidene difluoride (PVDF) membrane (Millipore). Tipe2 was detected using a specific antibody as previously described [48]. The following primary antibodies were used: anti-ERK and p-ERK, anti-Akt and p-Akt, anti-p-Smad and anti-Smad (CST), anti-c-Myc and anti-c-Ets-2; anti-β-actin (Proteintech). Immunoblotting was conducted by incubating the membranes with primary antibodies at 4 °C overnight followed by secondary antibodies (goat anti-rabbit Ig G or goat anti-mouse Ig G, 1:5000) conjugated with peroxidase for 1 h at room temperature. After washing, bound peroxidase activity was detected by the ECL detection system (ECL, F-cheiBIsi.6pro, DNR) using the Super Signal West Pico kit (Pierce Biotechnology). The images were analyzed using ImageJ software (National Institutes of Health, Bethesda, MD).

### Statistical analysis

All data were expressed as mean ± SEM (standard error of the mean). Statistical analysis was performed using GraphPad Prism 8 (GraphPad Software Inc. San Diego, CA). Significant difference between two groups was determined using an unpaired two-tailed Student’s *t*-test. *P* < 0.05 was considered as statistically significant.

## Supplementary information

Supplemental Mterial
