# Supplementary material for: The overexpression of Tipe2 in CRC cells suppresses survival while endogenous Tipe2 accelerates AOM/DSS induced-tumor initiation
Source: Cell Death Dis. 2021 Oct 26;12(11):1001. doi: 10.1038/s41419-021-04289-0 (PMC8548391; doi:10.1038/s41419-021-04289-0)
Supplement: Supplementary file 1 — Supplemental Mterial [file 41419_2021_4289_MOESM1_ESM.pdf]

Supplementary Figures

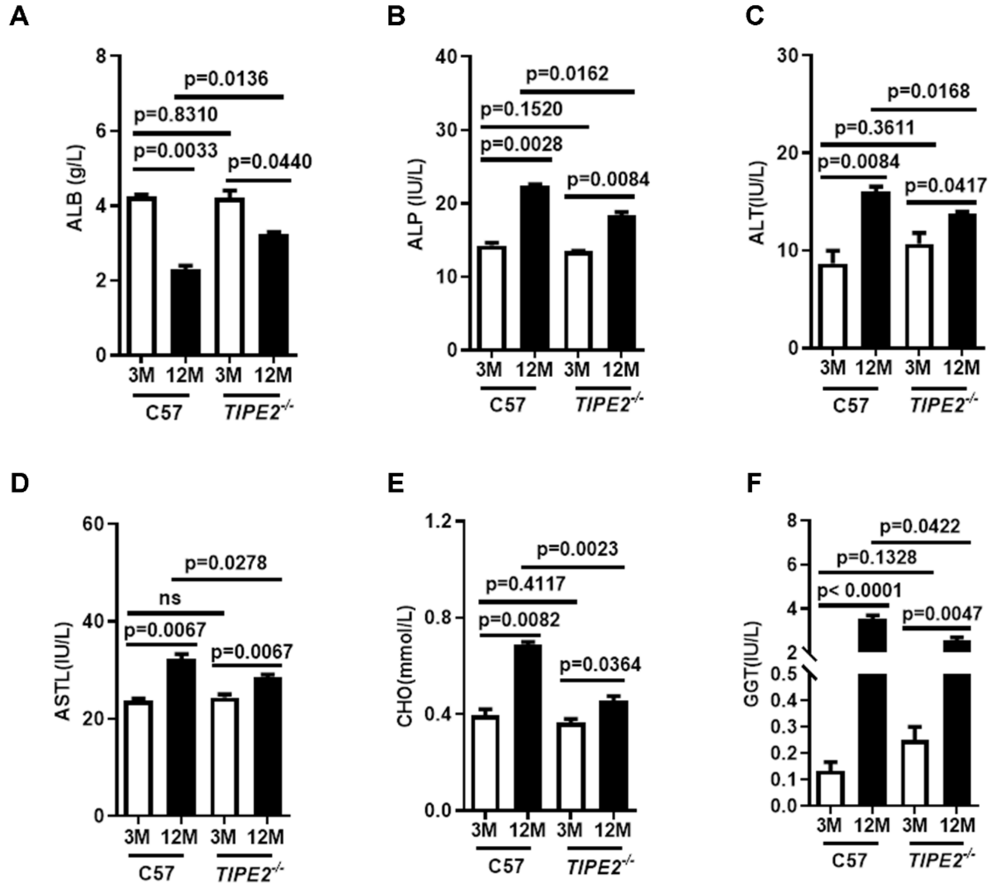

Figure S1 The serum levels of ALB (A), ALP (B), ALT (C), ASTL (D), CHO (E), and GGT (F) in *TIPE2* KO and matched WT mice with aging.

Data are representative of three independent experiments and expressed as means ± SEM. Significant difference between two groups was determined using an unpaired two-tailed Student's *t*-test.

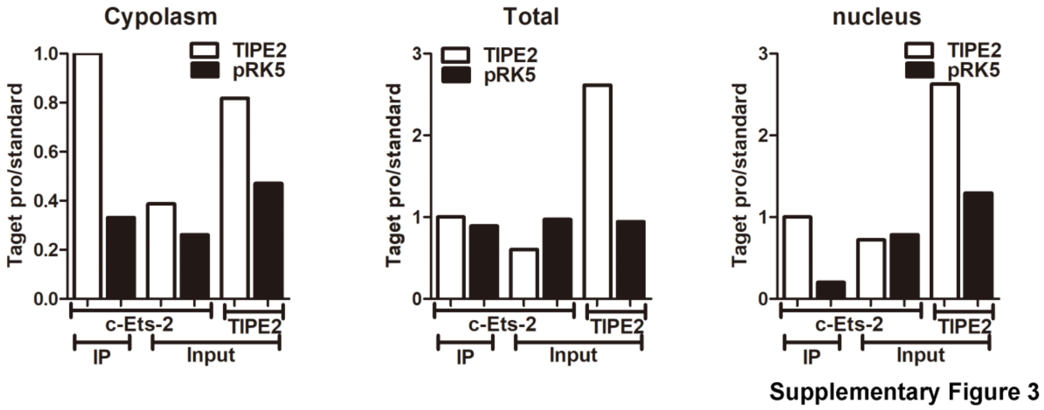

Figure S2 The quantification for Figure 2I.

The images were analyzed using ImageJ software.

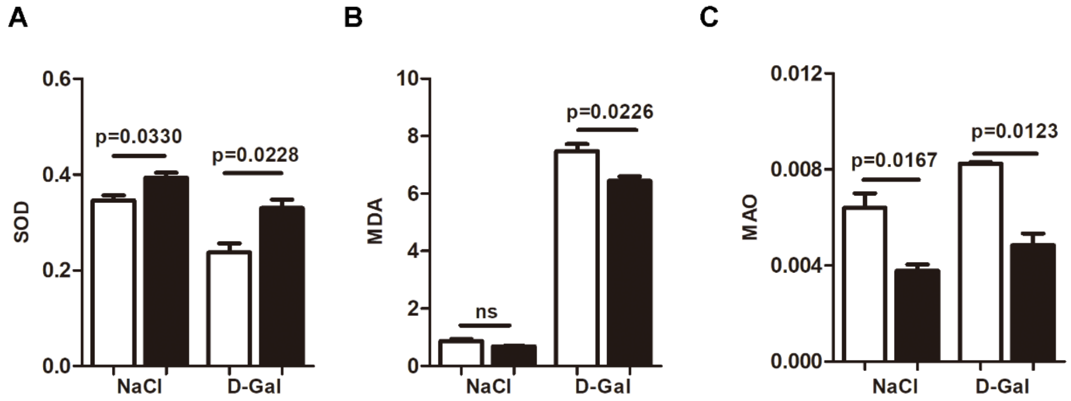

Figure S3 The detection of enzymes involved in oxidative stress in D-Gal induced TIPE2-deficient mice and WT.

Data are representative of three independent experiments and expressed as means ± SEM. Significant difference between two groups was determined using an unpaired two-tailed Student's *t*-test. $*P < 0.05$, $**P < 0.01$, $***P < 0.001$.

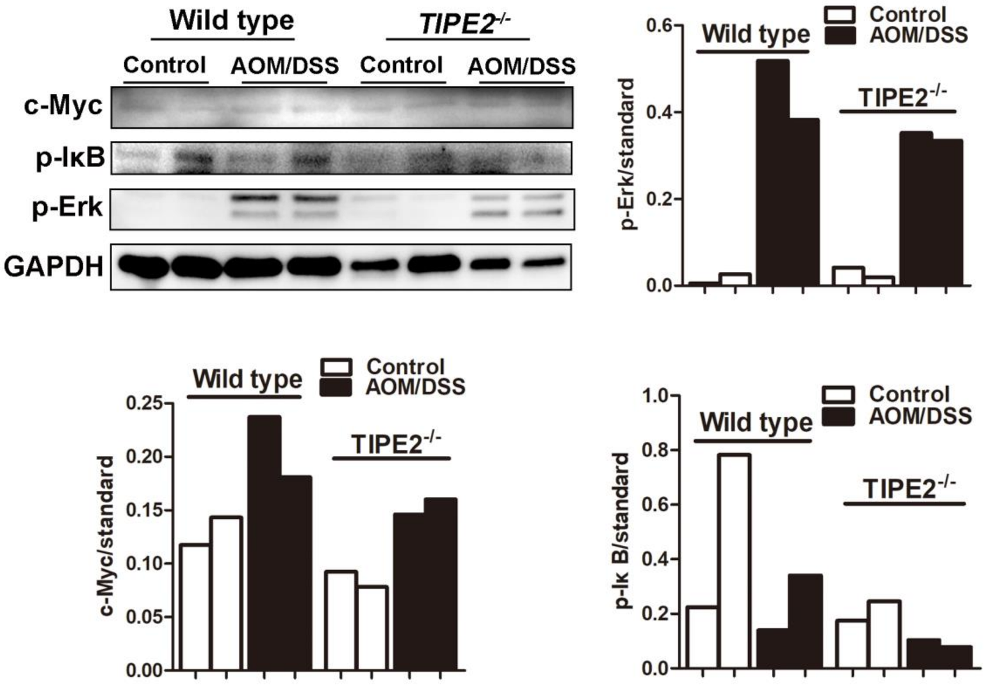

Supplementary Figure 4

Figure S4 The detection of c-Myc expression and NF-κB/ERK activation in AOM/DSS models.

The images were analyzed using ImageJ software.
